# The Diagnosis of Periodontal Disease*Read before the National Dental Association, Boston, Massachusetts, Aug. 23-27, 1920. Printed in *Journal N. D. A.*, Aug., 1921.

**Published:** 1921-09

**Authors:** Russell W. Bunting


### THE DIAGNOSIS OF PERIODONTAL DISEASE*

BY RUSSELL W. BUNTING, D.D.SC.,

Of all the diseases which lie within the realm of dental practice those which affect the tissues immediately surrounding the teeth are most imperfectly understood and are given the least attention. It is not that these diseases are unimportant, for by their ravages they accomplish as much if not more damage to the dental apparatus as do those which primarily affect the teeth. Nor does this apparent neglect arise from the fact that these diseases are uncontrollable or incurable, for of all oral affections there

*Read before the National Dental Association, Boston, Massachusetts, Aug. 23-27, 1920. Printed in *Journal N. D. A.*, Aug., 1921.

are none which may be so definitely and successfully prevented as are those which affect the periodontal tissues. Truly this is anomalous in that as a profession we should overlook or treat lightly a disease which is of so great significance and which, if we would, we might almost completely eradicate.

This attitude toward periodontal disease arises chiefly from the fact that these tissue disturbances are not recognized, or their significance is minimized until they have passed through the primary stages, in which they may be controlled, and have arrived at the more advanced stages, in which considerable tissue destruction has been accomplished and successful treatment is exceedingly difficult or impossible. As a result of their failure to obtain results in the treatment of these advanced cases, many practitioners have become disheartened and have lost faith in their ability to control periodontal disease.

The hope of controlling periodontal disease does not lie in the treatment of advanced cases but in the early recognition and correction of incipient forms of the disease which readily respond to preventive measures. These earlier stages consist of disturbances of circulation and nutrition in the periodontal tissues, which disturbances lead up to tissue destruction or metaplasia. If at this time the disturbing factors be removed and the normal circulation be reestablished the tissues will again receive their proper nourishment and their subsequent destruction will be avoided.

The prevention of periodontal disease is therefore very largely dependent on the early recognition of the departure of the tissues about the teeth from their normal physiological function and the diagnosis of the type of disturbance which has taken place in them. It is then to a discussion of the means of diagnosis of the various types of periodontal disease as related to the methods of their prevention that your attention is called at this time.

It should be recognized at the outset that the course of these diseases, like that of all other bodily affections, is the resultant of two factors, one the attacking force and the other the resistance of the tissues attacked. The first of these consists of various local irritants: (1) traumatic, as occlusion, food impactions, calculi, improperly adapted fillings, crowns, bridges, etc.; (2) chemical, as circulatory depositions of mercury, salts of uric acid, and other irritating substances; (3) bacterial, as streptococci, staphylococci, fusiform bacilli, etc. The effect that this first group of irritating substances may produce on the periodontal tissues is determined by the strength of their attack and by the type of resistance with which the local tissues combat them. This second factor, that of tissue resistance, is also a variable quantity. It is dependent on the type of circulation, density, and physiological function of the local tissues, which properties are in turn largely determined by the quality of general health and constitutional status of the individual. In the evaluation of each case of periodontal disease one must consider not only the local forms of irritation but also the general health of the individual as expressed in terms of local tissue resistance. Periodontal disease is a local disease inasmuch as its initial manifestations are confined to the tissues which surround the teeth, but the course of the disease, whether it be mild or severe, superficial or deep-seated, will be largely determined by the general health and resistive powers of the individual.

In view of the wide range of local and constitutional factors which may enter into the various cases of periodontal disease it will be recognized that these are extraordinarily complicated and involved processes. As each may be the product of a number of local and systemic factors, it follows that almost an infinite number of combinations may be formed by the harmful agencies which militate against the health and integrity of the periodontol tissues. Consequently this disease is not uniform or constant in its manifestations but presents many phases and variations during its course. Success in treating and controlling these conditions is dependent on the recognition of the most predominating factors in each case and the institution of measures which will either remove the determining causes or reduce them to a state of inefficiency. To accomplish this a close study must be made of the various cases which are presented and methods of diagnosis must be established by which the various types of periodontal disease may be recognized to the end that intelligent treatment may be instituted to control them.

Our means of diagnosis of periodontal diseases are limited to certain subjective signs or symptoms which may be noted in the tissues affected. Sometimes these symptomatic changes are in striking and vivid contrast to the normal tissues, so much that they compel the notice of even the casual observer. Not infrequently, however, pronounced pathological changes are produced in the tissues with but slight outward evidence of their presence. These latter often escape observation until they have seriously and irreparably injured the underlying tissues. It is very essential, therefore, that a definite routine method of examination be adopted in all cases by the application of which the health of the periodontal tissues may be rightly judged and the insidious as well as the conspicuous forms of disease discovered. For this purpose the following classification of tissue changes has been adopted: changes in color, changes in form, changes in density, changes in attachment to the necks of the teeth. These are supplemented by secondary or more advanced local symptoms as: loosening of the teeth, presence of pus, radiographic evidence, and a consideration of the general systemic health.

*Changes in color*—The normal color of the gum tissues varies in different individuals ranging from light to dark shades of pink, but in all cases *the color is uniform through-*

*out.* This color is the reflection of the hemoglobin in the capillaries, which lie immediately beneath the mucous membranes, while the depth of the color is determined by the quantity and quality of blood in the vessels. In full-blooded individuals the submucous circulation is filled with red blood which gives a deep pink or dark red color to the gum tissues. In contrast to these the anemic and under-nourished types have less blood in their peripheral circulation, the hemoglobin content is usually low, and the color of the gums is normally, for them, a light shade of pink. Thus we may read in the general gum colors many interesting and important facts concerning the general health and the character of the general circulation.

Any localized deviation from that uniform color characteristic of the individual is indicative of local changes in the circulation. Darker shades of red usually denote hyperemia, the depth of color depending on the severity of the circulatory disturbance. Deep red and blue are indicative of stasis and passive congestion resulting in an engorgement of the submucous tissues with venous blood. These localized changes in gum color should be a signal to the operator of danger, for, as a result of circulatory derangement, these tissues will suffer from a lack of nourishment and will lose their normal tone and resistive powers. It may also be noted in the vicinity of these color changes some form of irritant, either chemical, bacterial, or mechanical, may be found which is producing an active injury; upon removal the color of the tissues, as a rule, promptly returns to normal. By experience one may also read in these gum colors the types of protective reaction which are being put forth by the tissues to combat the injury. When the tissues are red and give evidence of strong inflammatory reaction the disease is largely superficial and the deeper tissues are involved only after a considerable lapse of time. Cases of this type respond readily to treatment and the tissues have a marked recuperative power. Conversely, when the gum colors are pale or dark blue the tissue disturbances are more deep-seated and their response to treatment is less favorable. Color changes therefore constitute a very important diagnostic factor in the discovery of periodontal disease and in the evaluation of local tissue reactions.

*Changes in form.*—Normally the gum contour about the teeth follows that of the edge of the enamel at the neck of the tooth, which it slightly overlaps. In the interproximate it extends up to the point of contact completely filling the space between the adjacent teeth. Deviations from the normal contours are of two types, namely shrinkage or retraction of the gum line and swelling or hypertrophy of the gum tissues. Examples of the former may be seen in the retraction of the buccal or lingual contours resulting from toothbrush abrasions or the atrophy of old age. So also are the gingival tissues retracted following deep involvement by periodontal disease. Swelling of the gum tissues about the teeth is indicative of a strong inflammatory reaction induced by some form of irritation and usually signifies a high degree of tissue resistance and recuperative power. When the tissues show a tendency to shrink and contract under injury it may be inferred that their resistance is low and that degenerative changes may easily be induced in them.

*Changes in density.*—The normal gum tissues are hard, firm and resistant to mechanical injuries. When in health the mucous membrane is tightly stretched over an underlying dense connective tissue which is supported by the alveolar process. This epithelium should be so tough that even under vigorous mastication or brushing of the teeth abrasion or hemorrhage will not occur. Soft and spongy gums, however, are easily torn and abraded and hemorrhage may be produced in the course of ordinary mastication or by slight injuries. When in this condition the gum tissues are never in health but rather are they subject to infection and may be severely injured by insignificant causes. Spongy gums are indicative of chronic passive congestion of the local tissues, which may arise from continued local irritation, from lack of gum massage, or from systemic or constitutional causes.

*The line of attachment.*—Normally the gum tissues are firmly attached about each tooth to the peridental membrane at the bottom of a shallow gingival crevice. When by exploration it is determined that this normal attachment has been disturbed and that a space exists between the root and surrounding tissues, it is evident that the underlying structures have been severely injured. Intensive inflammation and pronounced swelling may be present in the superficial and gingival tissues, but as long as the line of attachment about the root remains normal, serious disturbance of the underlying tissues has not occurred. Therefore by careful exploration of all gingival crevices one may determine by their depth whether the case under observation is superficial or deep-seated in type. This is indeed the most important and most certain method of diagnosing periodontal disease.

*Loosening of the teeth.*—It is only in the more advanced and severe cases that the teeth become loosened in their sockets. Since the lesions, as a rule, are confined to but one or two sides of the teeth and tend to extend apically along the lateral surfaces of the roots rather than about them, deep lesions may be produced about teeth which still possess sufficient peridental attachment to hold them securely in position. When by reason of the extension of the disease one or more of the teeth become loose it may be inferred that the condition is very serious and the possibility of successful treatment is doubtful. When there is only a moderate degree of movement and when by radiographic evidence it is determined that at least the apical half of the root is imbedded in bone, if other conditions permit, treatment may be attempted. But when on percussion the tooth plays up and down or may be rotated in its socket, and the radiographic findings show that the surrounding bone has been destroyed, treatment is usually contra-indicated.

*Pus.*—The presence of pus has been used by many as an important and specific means of diagnosing periodontal disease. But pus is by no means a specific feature of this disease inasmuch as a large majority of its lesions have no visible purulent discharge. The only information that may be derived from the discovery of pus is that the predominating organisms are purulent in type and that against them the tissue reactions are of good order. Instead of being a symptom of grave significance, pus is an indication that the tissues are combatting the infection and perhaps controlling it and that in view of the active local reaction the prognosis of treatment of such cases is favorable.

*Radiographic findings.*—There are many radiographers who claim that they are able to diagnose all stages of periodontal disease by the radiograph, but in our experience this form of diagnosis is accurate and valuable for only one phase of the process, namely, the extent of bone destruction. In all other questions regarding the extent of involvement one may easily be misled by radiographic evidence; for instance, it is difficult and often impossible by these means alone to differentiate between an active and a healed lesion of the disease. An opinion, therefore, should never be formulated from the evidence of a radiographic film alone, but rather should all other means of diagnosis be employed in which the radiograph is but one factor. From the radiograph a more or less accurate conception of the amount of bone destruction may be obtained. In this also there is a possible source of error in that the destruction of the interproximate bone may take place to produce a hole or cusp-shaped depression between two adjacent teeth, while the labial and lingual plates are still intact, in which case the exact status of the osseous lesion is not easily determined by the radiograph. So also the destruction of bone on either labial or lingual aspects of the tooth may not be apparent when the opposite bony walls are unaffected, as the shadow of the healthy portion obscures the outlines of that which is diseased. Too much dependence, therefore, should not be placed on the radiograph in the diagnosis of periodontal disease but rather should it be employed only as an auxiliary and a subordinate factor in the whole chain of evidence.

*General considerations.*—By means of the foregoing methods of diagnosis fairly definite information may be obtained regarding the local conditions, but whenever it is determined in this manner that the periodontal tissues are diseased the examination should be extended to include the general state of health of the individual. In severe cases secondary effects may often be noted in the general health, while in the incipient forms of the disease underlying causative factors may frequently be recognized which constitute active or predisposing factors in the case. For example, periodontal disturbances commonly occur in conjunction with malnutrition, intestinal stasis, uric acid diathesis, and many other general disorders, so much so that an interrelationship between the local and general disturbances is often very apparent. Too much stress cannot be laid on the importance of considering the patient as a whole. When certain types of cases are treated solely from a local standpoint the benefits of such treatment are but transitory and the disturbances tend to recur. If, however, the underlying systemic factors are recognized and corrected the local conditions may be successfully controlled. The diagnosis of all advanced or persistent cases of periodontal disease should include a complete physical examination of the patient, which procedure should also be followed in the incipient and milder forms of the disease whenever possible.

The foregoing methods of diagnosis are presented, not as an original of infallible means or recognizing periodontal disease but rather are they intended as a simple workable scheme which, if adopted as a routine part of everyday practice, will enable the operator to discover many of the incipient forms of this disease which heretofore he may have unwittingly overlooked. When the habit of observation has been developed to the point that he may read these gum conditions at sight and when he recognizes how prevalent these incipient periodontal disturbances are then will the operator be impelled to institute prophylactic measures for such cases which will check these diseases in their inception and prevent their more serious sequelae. Thus may we most effectively control periodontal disease.

